# Waste biorefinery towards a sustainable circular bioeconomy: a solution to global issues

**DOI:** 10.1186/s13068-021-01939-5

**Published:** 2021-04-07

**Authors:** Hui Yi Leong, Chih-Kai Chang, Kuan Shiong Khoo, Kit Wayne Chew, Shir Reen Chia, Jun Wei Lim, Jo-Shu Chang, Pau Loke Show

**Affiliations:** 1 Key Laboratory of Biomass Chemical Engineering of Ministry of Education, College of Chemical and Biological Engineering, Zhejiang University, Hangzhou, 310027 China; 2 Department of Chemical Engineering and Materials Science, Yuan Ze University, No. 135, Yuan-Tung Road, Chungli, Taoyuan, 320 Taiwan; 3 Department of Chemical and Environmental Engineering, Faculty of Science and Engineering, University of Nottingham Malaysia, Jalan Broga, 43500 Semenyih, Selangor Darul Ehsan Malaysia; 4 School of Energy and Chemical Engineering, Xiamen University Malaysia, Jalan Sunsuria, Bandar Sunsuria, 43900 Sepang, Selangor Darul Ehsan Malaysia; 5 Department of Fundamental and Applied Sciences, HICoE-Centre for Biofuel and Biochemical Research, Institute of Self-Sustainable Building, Universiti Teknologi PETRONAS, 32610 Seri Iskandar, Perak Darul Ridzuan Malaysia; 6 Department of Chemical Engineering, National Cheng Kung University, Tainan, 701 Taiwan; 7 Department of Chemical and Materials Engineering, College of Engineering, Tunghai University, Taichung, 407 Taiwan; 8 Research Center for Smart Sustainable Circular Economy, Tunghai University, Taichung, 407 Taiwan

**Keywords:** Bio-lipids, Biopolymers, Circular bioeconomy, Waste biorefinery, Wastewater bioremediation

## Abstract

Global issues such as environmental problems and food security are currently of concern to all of us. Circular bioeconomy is a promising approach towards resolving these global issues. The production of bioenergy and biomaterials can sustain the energy–environment nexus as well as substitute the devoid of petroleum as the production feedstock, thereby contributing to a cleaner and low carbon environment. In addition, assimilation of waste into bioprocesses for the production of useful products and metabolites lead towards a sustainable circular bioeconomy. This review aims to highlight the waste biorefinery as a sustainable bio-based circular economy, and, therefore, promoting a greener environment. Several case studies on the bioprocesses utilising waste for biopolymers and bio-lipids production as well as bioprocesses incorporated with wastewater treatment are well discussed. The strategy of waste biorefinery integrated with circular bioeconomy in the perspectives of unravelling the global issues can help to tackle carbon management and greenhouse gas emissions. A waste biorefinery–circular bioeconomy strategy represents a low carbon economy by reducing greenhouse gases footprint, and holds great prospects for a sustainable and greener world.

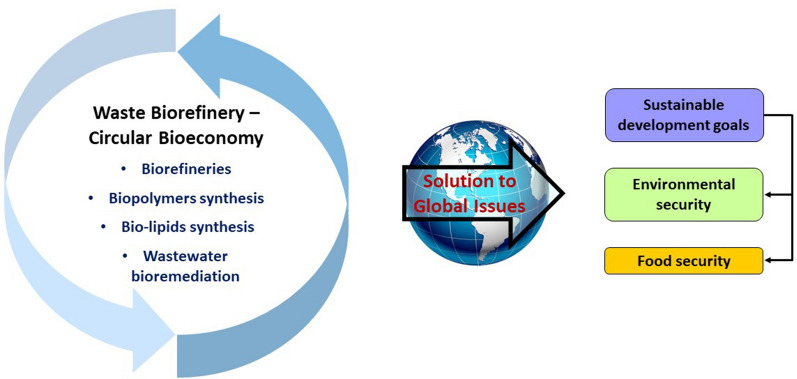

## Background

According to the Worldometer, the current world population records 7.8 billion people as of August 2020, and it is projected to be 10 billion people in 2057 [1]. The high annuals of world population growth are tackling pressing challenges on global issues pertaining to environmental problems and food security which affect the Sustainable Development Goals (SDGs). Particularly, such environmental concerns like pollutions, climate change, global warming, waste disposal and natural resource reduction have increased at an alarming rate, and these concerns are mostly a consequence of uncontrolled detrimental activities by human being on our Mother Earth [2]. For example, the extensive exploitation of petroleum or fossil fuel resources to produce energy, chemicals and synthetic materials not only causes the depletion of natural non-renewable resources but also impact the high releases of greenhouse gases (GHGs) emission, which affects the environment dramatically [3]. In consequence, these global problems need an imperative solution where circular bioeconomy can play the major role in which a low carbon economy will definitely help to resolve these issues, especially on the climate change through limiting global warming by 1.5 °C henceforth [4–7].

The term circular bioeconomy, also known as bio-based circular economy, is an integrated concept of circular economy and bioeconomy. In other words, it denotes the cascading use of biomass from biological resources into a systemic approach for economic development. A circular bioeconomy offers an efficient utilisation of biomass which include wastes and side streams for the sustainable production of high value-added products (e.g., food, biomaterials, feed and bioenergy). The benefits of circular bioeconomy includes: (1) improved resource and eco-efficiency, (2) lower GHGs footprints, (3) reduced reliance of fossil resources and (4) valorisation of side and waste materials from numerous sources such as agro-industrial aquaculture and fishery. This concept focuses on the idea of recycling, reuse, remanufacture and maintaining a sustainable manufacturing process to generate useful bioproducts. Hence, circular bioeconomy can be reflected as a low carbon economy since it exhibits the potential on developing a sustainable and greener environment [7–9].

Biorefining is among one of the most primary facilitating strategies of the bio-based circular economy that closes the loop of fresh or raw resources, water, minerals and carbon. It can be defined as the sustainable bioprocesses that efficiently utilise biomass resources for the production of various marketable products and metabolites (e.g., carbohydrates, proteins, lipids, bioactive compounds and biomaterials) [10]. Furthermore, waste biorefinery receives as much interest or even higher as it represents a decent waste management approach [2, 11, 12]. Bioprocesses utilising waste resources to produce biomaterials and biofuels can greatly elude fossil resources as the production feedstock and this prevents the natural resources from complete depletion. This approach does not only sustains the energy–environment nexus but also protects the environment by mitigating the carbon footprints (i.e., GHGs emission from burning fossil resources) [13]. Moreover, these bioprocesses can be incorporated with other management facilities such as wastewater treatment [14]. Biopolymers (e.g., polyhydroxyalkanoates and polyhydroxybutyrates) and biofuels (e.g., biodiesel, bioethanol, biohydrogen and biogas) are eco-friendly bioproducts that can be produced from various bioprocesses using a wide selection of renewable feedstocks [15]. As for the biofuels production, metabolites like lipids and carbohydrates are first synthesised through bioprocesses which are then followed by further processing on the metabolites into bioenergy. On the other hand, bioprocesses that involves fermentation can directly synthesize biopolymers [e.g., polyhydroxyalkanoates (PHAs)] [8, 16, 17].

**Fig. 1:**
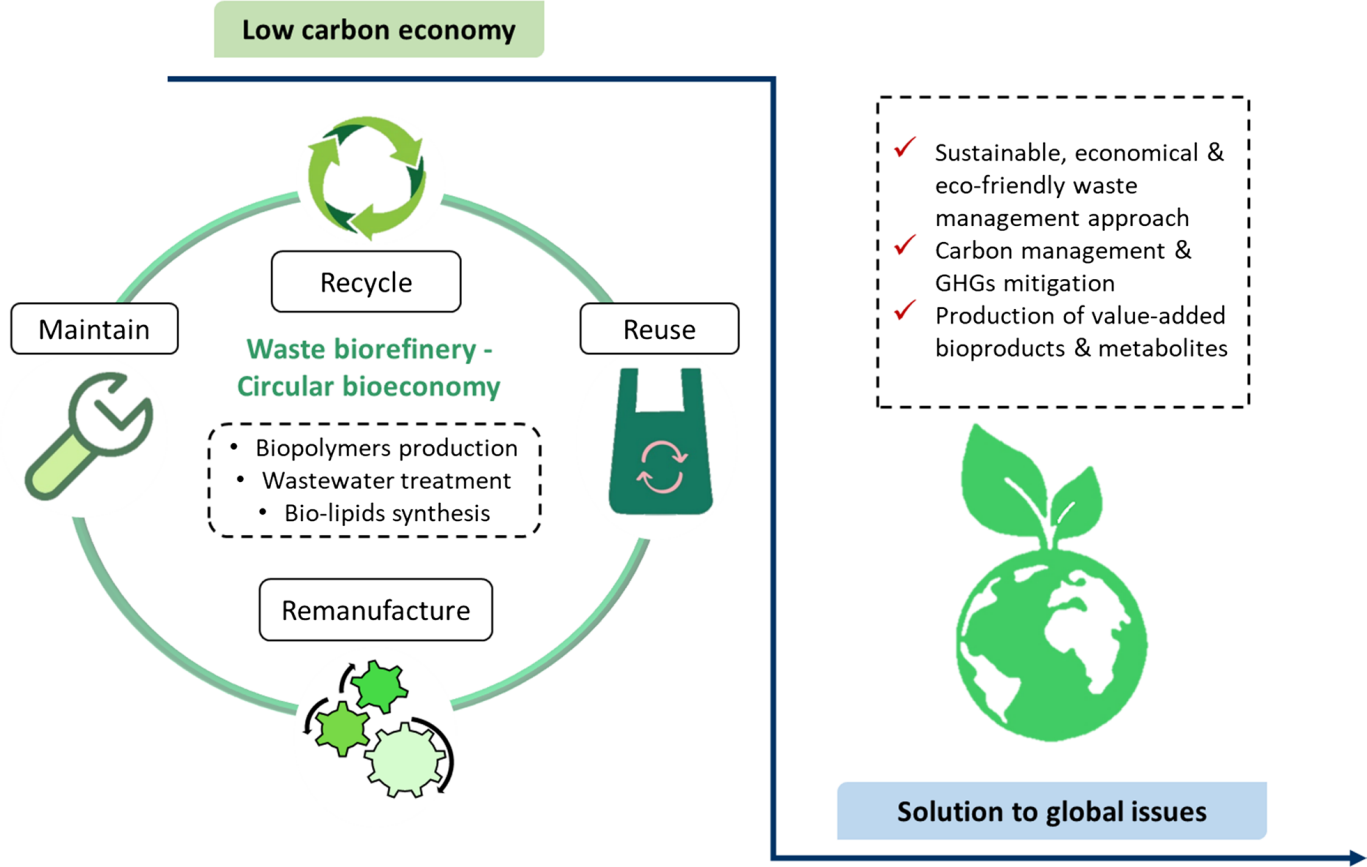
Schematic view delineates the outline of this review article. The concept of low carbon economy involves recycling, reusing, remanufacturing and maintaining existing processes

Taking the above issues into consideration, this review article aims to evaluate the waste biorefinery advocating toward a circular bioeconomy. Several case studies on the bioprocesses regarding the waste biorefinery to produce biopolymers and bio-lipids have been reviewed. Besides that, the investigation of bioprocesses incorporated with wastewater treatment have been analysed and well discussed. The final section of this review comprehensively evaluated on how the integrated concept of waste biorefinery and circular bioeconomy can contribute towards resolving the global issues especially on the environmental concerns and food security (see Fig. 1). Circular bioeconomy is crucial and possesses vast potential towards a sustainable green world. Respectively, waste biorefinery holds great prospective for the forthcoming circular bioeconomy.

## Waste biorefinery promoting a circular bioeconomy

Petroleum or fossil fuel is a natural resource which has been the utmost important production feedstock for energy (e.g., transportation fuels) and synthetic materials (e.g., plastics and chemicals) for decades. However, they are non-renewable and possess environment-threatening features which causes climate change by the emission of GHGs mainly carbon dioxide (CO_2_) to the atmosphere. These environmental issues have raised the global awareness and there are a great deal of researches on carbon mitigation and adaptation [18]. Shifting towards a waste biorefinery model from a petroleum refinery model indicates a great effort on the carbon management and GHGs mitigation. Waste biorefinery involves in the establishment of a sustainable circular bioeconomy based on the philosophy of recycle, reuse, remanufacture and maintaining by shifting from a linear economy according to the principle of take, make and dispose [7, 19, 20].

Bioprocesses using waste materials which consists of municipal solid and liquid waste to produce value-added bioproducts and metabolites regarded as waste-to-treasure has received increasing attention as the products produced are renewable and display environmental benign biodegradability characteristics. The bioprocessing of waste biorefinery on the production of biopolymers and bioenergy does not only addresses the energy and environmental security concerns, in fact it signifies a better management of waste streams. It is an eco-friendly and economically sound platform as the production feedstock is sustainable and low in cost [21]. Various kind of waste materials such as food waste [22], side stream from industries (e.g., paper and pulp industry, beer and wine industry, starch and juice industry), agro-industrial by-product [23, 24], forest and agriculture waste, lignocellulosic material [25] as well as wastewater or sludge [26], have been efficiently valorised into useful and marketable bio-based products [8, 20, 27]. Several case studies on the bioprocesses using waste for biopolymers and bio-lipids to be further converted into biofuels production as well as bioprocesses for wastewater treatment are next discussed in the following sub-sections.

### Bioprocesses with waste for biopolymers synthesis

Shifting to a more eco-benign environment, PHAs which represent the green biopolymers have captured tremendous attention from both the industry and scientific community driven by the need to replace conventional petroleum-derived non-degradable polymers or plastics. They possess enormous inherent properties such as insolubility in water, non-toxicity, biodegradability, biocompatibility, piezoelectricity and thermoplasticity, and hence showing potential as substitute of petrochemical plastics (e.g., polypropylene and polystyrene) [28]. PHAs, a type of linear polyesters of hydroxyalkanoates (HAs), can be produced through microbial fermentation with renewable resources like waste and side streams [29, 30]. Usually, they are accumulated as intracellular carbon and energy storage compounds in the culture under limited growth conditions with excess carbon sources. PHAs can be divided into three groups, which are based on the number of carbon atom: SCL-PHAs (short chain length PHAs; 3–5 carbon atoms), MCL-PHAs (medium chain length PHAs; 6–15 carbon atoms) and LCL-PHAs (long chain length PHAs; ˃ 15 carbon atoms). There are many varieties of PHAs, for instance, poly(3-hydroxybutyrate) (PHB), poly(3-hydroxyvalerate) (PHV) and poly(3-hydroxybutyrate-*co*-3-hydroxyvalerate) (PHBV). PHAs have excellent potential applications in industrial, agricultural, domestic and medical field. For example, they are widely applied in tissue engineering as supportive scaffolds, in packaging industries and in drug delivery as nanoparticles [31–34].

Reutilisation of waste resources using bioprocesses to produce biomaterials like PHAs gains increasing importance for environmental and socio-economic reasons. This initiative supports an eco-friendly campaign besides reducing the production cost. As an example, the utilisation of CO_2_ and valeric acid in the *Cupriavidus necator* DSM 545 fermentation to tailor microbial PHBV. The carbon fixation and utilisation system have been successfully applied to produce the microbial bioplastic, and this will achieve a low carbon economy [30]. Another study by Koller et al. [35] reported that the valorisation of surplus agricultural waste materials into cheap and good carbon, nitrogen and phosphorus sources with a highly osmophilic strain on the production of PHA. These materials included the hydrolysation of whey permeate and glycerol liquid phase as carbon source, as well as meat and bone meal as nitrogen and phosphorus sources. These hydrolysed waste resources utilised in the fermentation process can reduce the production cost of PHA. Besides that, the assimilation of agro-industrial oily waste with *Pseudomonas aeruginosa* 42A2 (NCIB 40045) fermentation for PHA production was studied by Fernández et al. [36]. Waste frying oil and waste-free fatty acids from soybean oil were used as the carbon source on the microbial fermentation, and successfully accumulated PHA of 29.4% and 66.1%, respectively.

Other than that, Yu and team studied the PHAs production using starchy wastewater with *Alcaligenes eutrophus* (ATCC17699) cultivation [37]. The research employed a two-step process of microbial acidogenesis and acid polymerisation. The organic waste firstly undergoes acidogenesis to produce volatile fatty acids (VFAs) (e.g., acetic, propionic, and butyric acid) under anaerobic conditions, and was subsequently used to produce microbial PHAs. Also, dairy industrial waste, rice bran and sea water were employed by RamKumar Pandian et al. to synthesise PHB with *Bacillus megaterium* SRKP-3 fermentation. The maximum PHB concentration (11.32 g/L) was achieved in the microbial culture with the dairy waste [38]. Furthermore, the utilisation of food waste, acidogenic effluents and waste glycerol for the production of PHAs were reported previously by Venkateswar Reddy and Venkata Mohan [39] and Cavalheiro et al. [40], respectively. The former study showed that the microbial culture with acidogenic effluents (fermented food waste) accumulated a higher PHA (39.6%) compared to that of with unfermented food waste (35.6%). The study demonstrated the production of biohydrogen along with biopolymer using microbial fermentation process with fermented food waste. Whereas, the latter study valorised waste of crude glycerol by-product of biodiesel production with *Cupriavidus necator* DSM 545 fermentation to produce PHB (50% of PHB, w/w). To date, there are still many ongoing researches on the biopolymers production by valorisation waste streams with bioprocesses [41–44].

Bacterial cellulose (BC) is an alternative green biopolymer which has also been extensively studied by the scientific community. BC is a natural nano-polymer that displays numerous interesting characteristics, including higher degree of polymerisation, higher tensile capability, higher crystallinity, higher purity as well as good water absorbing and holding capacity, in addition to the good biological adaptability [45, 46]. These inherent properties have prompted the wide applications of BC in various fields include pharmaceutical, biomedical and food [47]. Many researchers have valorised different type of wastes with bacterial cultivation to produce BC, for instance, waste fibre sludge [48], waste from beer industry [49], black strap and brewery molasses [50], wastewater of candied jujube-processing industry [51], corn steep liquor [52], sweet lime pulp waste [53] and many more [47]. Lin et al. [49] studied the cultivation of *Gluconacetobacter hansenii* CGMCC 3917 with only waste beer yeast as the nutrient and carbon sources for synthesis of BC, and a promising result was obtained. The bacterial culture achieved from the optimised waste beer yeast hydrolysate (treated by a two-step pre-treatment that incorporated ultrasonication and mild acid hydrolysis as well as optimisation of sugar concentration) resulted in a higher BC yield and demonstrated good physicochemical features (i.e., holding capacity, release rate and absorption rate of water) compared to that of using untreated waste beer yeast and conventional chemical media. Table 1 shows the various bioprocesses utilising waste materials for the biopolymers synthesis.

**Table 1:** Several bioprocesses using waste for biopolymers production

Microbial strain(s)	Waste material(s)	Culture mode	Results	References
Production of PHAs				
* Alcaligenes eutrophus* (ATCC17699)	Starch wastewater	A two-step process of microbial acidogenesis and acid polymerisation	PHA produced using VFAs 55 g of PHA per 100 g total organic carbon	[37]
* Bacillus megaterium* SRKP-3	Dairy waste	Fed-batch cultivation	PHB concentration = 11.32 g/L	[38]
* Burkholderia sacchari* DSM 17165	Wastepaper	Fermentation process	PHB content = 44.2% (using wastepaper hydrolysate as carbon source)	[43]
* Cupriavidus necator* DSM 545	CO_2_	Mixotrophic fermentation	PHBV content of 60% Represented a CCU strategy	[30]
* Cupriavidus necator* DSM 545	Crude glycerol	Fed-batch cultivation	PHB concentration = 38.1 g/L PHB/cell dry weight = 50% (w/w)	[40]
* Haloferax mediterranei*	Macroalgae biomass hydrolysates (macroalgae-derived carbohydrates; carbon source)	Fermentation process	Promising PHA production feedstock: *Ulva* sp. PHA concentration of 2.2 ± 0.12 g/L Sustainable PHA production using sea agriculture	[44]
* Halomonas* species	Waste frying oil (carbon source)	Fermentation process	*Halomonas hydrothermalis* with γ-Butyrolactone accumulated 2.26 g/L of PHA PHB producer: *Halomonas neptunia* CCM 7107 and *Halomonas hydrothermalis* CCM 7104 The *Halomonas hydrothermalis* was capable to accumulate PHV to form PHBV (culture supplemented with valerate)	[41]
* Pseudomonas aeruginosa* 42A2 (NCIB 40045)	Waste frying oil Waste-free fatty acids from soybean oil	Aerobic fermentation	PHA content = 29.4% (using waste frying oil as carbon source) PHA content = 66.1% (using waste-free fatty acids from soybean oil as carbon source)	[36]
A highly osmophilic strain	Hydrolysed whey permeate (by-product from cheese industry) Glycerol liquid phase (by-product from biodiesel production using plant oils and tallow) Meat and bone meal	42-L bioreactor fermentation system	PHA concentration = 5.5 g/L (using hydrolysed whey permeate as carbon source) PHA concentration = 16.2 g/L (using glycerol liquid phase as carbon source) PHA concentration = 5.91 g/L (using glycerol liquid phase as carbon source as well as meat and bone meal as nitrogen and phosphorus source)	[35]
Mixed microbial culture: species not mentioned specifically	Dairy waste (deproteinised cheese whey wastes)	A two-step bioprocess of dark fermentation and mixed microbial cultivation	Two bioproducts produced: biohydrogen and PHA Concentrated cheese whey permeate: H_2_: 1.93 mol H_2_ mol^−1^ sugars and 55.1 ± 1.3% g PHA g^−1^ volatile suspended solids Second cheese whey: H_2_: 1.37 mol H_2_ mol^−1^ sugars and 62.0 ± 4.5% g volatile suspended solids	[42]
Mixed microbial culture: species not mentioned specifically	Food waste (or known as unfermented food waste) Acidogenic effluents (from biohydrogen production; or known as fermented food waste)	Aerobic mixed cultivation	PHA content = 39.6% (using acidogenic effluents as substrate) PHA content = 35.6% (using food waste as substrate) PHA production in the form of PHBV; higher fraction of PHB than PHV Two bioproducts produced: biohydrogen and PHBV	[39]
Production of BC				
* Acetobacter xylinum* CGMCC 2955	Wastewater of candied jujube-processing industry	Fermentation process	BC productivity = 0.375 g/L/day	[51]
* Komagataeibacter europaeus* SGP37	Sweet lime pulp waste	Static batch cultivation	BC yield = 6.3 g/L	[53]
* Gluconacetobacter hansenii* CGMCC 3917	Waste beer yeast (hydrolysate obtained through a two-step pre-treatment)	Fermentation process	The highest BC yield = 7.02 g/L (using waste beer yeast hydrolysate treated by ultrasonication and mild acid hydrolysis as well as optimisation of sugar concentration) BC produced shows good physicochemical features (i.e., holding capacity, release rate and absorption rate of water)	[49]
* Gluconacetobacter hansenii* UCP1619	Corn steep liquor	Static cultivation	BC produced up to 73%	[52]
* Gluconacetobacter xylinum* ATCC 23768	Black strap molasses Brewery molasses	Fermentation process	BC yield = 3.05 g/L (using black strap molasses) BC yield = 1.78 g/L (using brewery molasses)	[50]
* Gluconacetobacter xylinus* ATCC 23770 (a bacterium used to produce BC) and *Trichoderma reesei* C-30 (a filamentous fungus used to produce enzyme)	Waste fibre sludge (derived from pulp mills and lignocellulosic biomass)	Sequential fermentation process	Co-production of BC and cellulase using fibre sludge hydrolysates Fiber sludges from sulfate produce 11 g/L of BC Fiber sludges from sulfite produce 10 g/L of BC	[48]

### Bioprocesses with waste for bio-lipids synthesis

The bioprocessing of waste contributes to both the production of green biopolymers and accumulation of bio-lipids. Production of microbial lipids using low-cost substrates from waste materials has attained much attention from both the industry and research areas as the alternative feedstock for biofuels production, health food supplements and oleo-chemical industries. Oleaginous microorganisms such as yeasts, cyanobacteria, algae, some bacteria, and fungi can accumulate significant amount of lipids of their body weight (~ 20–80%) [54]. The microbial oils are safe-to-use, non-toxic and biodegradable, whereby their industrial applications do not depend on petroleum-based chemicals. These features sustenance a greener environment for the society, and can help to alleviate several global issues [23, 55–59]. Nowadays, many researchers focused on the bioprocesses utilising waste materials for microbial lipids production [60, 61]. For instance, Fontanille et al. [56] reported the feasibility of simultaneous bio-valorisation of VFAs (e.g., acetic, propionic and butyric acid) and glycerol as the carbon sources for oleaginous yeast *Yarrowia lipolytica* MUCL 28849 culture to generate microbial lipids. These carbon sources are inexpensive and can be easily obtained from industries as by-product or waste. Similarly, Gong et al. studied the conversion of acetic acid waste into microbial lipids by cultivating *Cryptococcus curvatus* ATCC 20509 under various culture modes, and promising yeast-derived lipids yields were attained [62].

In addition, Huang et al. [63] and Xavier et al. [64] studied the valorised acetic acid and hemicellulose hydrolysate, respectively, on the production of yeast-derived lipids. The former study utilised 4–20 g/L acetic acid as the sole carbon source with *Rhodosporidium toruloides* AS 2.1389 culture to synthesise lipids of approximately 38.6–48.2%, while the latter study employed hemicellulose hydrolysate from sugarcane bagasse to cultivate *Lipomyces starkeyi* DSM 70296, and lipid content of 26.1–26.9% was obtained. Besides, Lopes et al. [65] reported the production of microbial lipids and some useful metabolites such as citric acid and lipase through cultivation of *Yarrowia lipolytica* W29 (ATCC 20460) with pork lard. Pork lard is an animal fat which is rarely used in food preparation, as its consumption causes vascular and heart diseases, and hence it is normally regarded as waste. This study revealed the possible usage of waste from meat processing industries for microbial oils synthesis.

Microalgae oil has gained high popularity in industrial applications such as biodiesel and health food supplements [66, 67]. Microalgal-derived biodiesel has excellent properties like low viscosity and represents as a carbon–neutral renewable fuel which benefits the environment and should be used to replace fossil fuels. Moreover, microalgae oil also contains polyunsaturated fatty acids (PUFAs) which can be further processed into health food supplements [68, 69]. Valorisation of waste into microalgae bioprocessing represents a greener and cost-effective circular bioeconomy approach. In this regard, Hong et al. [70] suggested that the empty palm fruit bunches can be a potential source for the production of microalgal lipids that contain significant amount of docosahexaenoic acid (DHA). DHA (C22:6*n *− 3) is an omega-3 PUFA that plays a vital role in brain and eye development. Another study by Chiranjeevi and Venkata Mohan [71] reported an integrated process of acidogenic fermentation and microalgae culture using wastewater to produce lipids. Two types of fermented effluents, include fermented distillery wastewater and fermented dairy wastewater, were employed in the cultivation with different culture modes (i.e., fermented distillery wastewater: mixotrophic culture and fermented dairy wastewater: both hetero- and mixotrophic culture).

Furthermore, microalgae *Chlorella vulgaris* FACHB-31 cultured with landfill leachate in membrane photobioreactors to produce bio-lipids was reported by Chang et al. [72]. The bio-lipids produced exhibited good combustion properties by owning low linolenic acid content (8.32%) and high cetane number (60.96%). A study by Nguyen et al. [73] also reported the utilisation of wastewater in the microalgae cultivation for lipids production. Seafood wastewater effluent was used to culture *Chlorella vulgaris* SAG 211-19, and lipid content of 32.15% was successfully produced. Much research efforts are being placed to valorise waste on microalgae cultivation to generate useful bioproducts [74–79]. Table 2 shows numerous bioprocesses using waste materials to synthesise microbial lipids. Collectively, assimilation of industrial by-product or waste biorefinery could be a good choice to turn the unwanted substances into useful product such as biopolymers and bio-lipids which represents a sustainable and economical waste management approach.

**Table 2:** Several bioprocesses using waste for bio-lipids production

Microbial strain	Waste material(s)	Culture mode(s)	Results	Reference(s)
Yeast cultivation				
* Cryptococcus curvatus* ATCC 20509	Acetic acid	Flask culture, 3-L stirred-tank bioreactor, continuous culture with nitrogen-rich condition at a dilution rate of 0.04 h^−1^	Lipid content = 73.4%, 49.9%, 56.7%, respectively	[62]
* Lipomyces starkeyi* DSM 70296	Hemicellulose hydrolysate (from sugarcane bagasse)	Flask culture, batch bioreactor culture	Lipid content = 26.9%, 26.1%, respectively	[64]
* Rhodosporidium toruloides* AS 2.1389	Acetic acid	Batch culture with 20 g/L acetic acid, sequencing batch culture with 4 g/L acetic acid	Lipid content = 48.2%, 38.6%, respectively	[63]
* Yarrowia lipolytica* MUCL 28849	VFAs (acetic, propionic and butyric acid) and glycerol	Two-stage fed-batch bioreactor fermentation	Lipid content = ~ 40% Lipid concentration = 12.4 g/L	[56]
* Yarrowia lipolytica* W29 (ATCC 20460)	Pork lard	Batch culture	Lipid content = 58%	[65]
Algal cultivation				
* Aurantiochytrium limacinum* SR21	K_2_HPO_4_-waste feedstock	Lab-scale flask culture (100 mL)	Lipid content = 8.29% DHA production = 128.81 mg/L	[76]
* Aurantiochytrium* sp. KRS101	Empty palm fruit bunches	5-L fermenter	Lipid content = 36.3% Lipid concentration = 12.5 g/L DHA concentration = 5.4 g/L	[70]
* Chlorella sorokiniana* CY-1	Palm oil mill effluent	Bioreactor fermentation process	Biomass concentration = 2.12 g/L Lipid content = 11.21% (using acid-heat pretreated 30% (v/v) palm oil mill effluent)	[74]
* Chlorella sorokiniana* 211-32	Acetate-rich oxidised wine waste lees	Fed-batch mixotrophic culture	Biomass concentration = 11 g/L Lipid content = 38%	[77]
* Chlorella vulgaris* FACHB-31	Biological effluent of landfill leachate	Membrane photobioreactor fermentation system	Bio-lipids produced displayed good combustion properties Low linolenic acid content (8.32%) and high cetane number (60.96%)	[72]
* Chlorella vulgaris* SAG 211-19	Seafood wastewater effluent	Bioreactor fermentation process	Lipid content = 32.15%	[73]
* Scenedesmus* sp. R-16	Starch-rich food waste	A two-stage process: dark fermentation and microalgal culture (bioreactor culture)	Biohydrogen yield = 1643.5 mL/L Lipid yield = 515.6 mg/L	[79]
* Scenedesmus* sp.	Agricultural biomass waste (corn cob and stalk, rice and wheat straw)	A two-stage process: dark fermentation and microalgal culture (batch culture)	Corn stalk was the best fermentation feedstock for biohydrogen production Biomass concentration = 1461.1 mg/L Lipid content = 35.2% (using corn stalk as substrate)	[78]
* Tetradesmus obliquus* AARL G022	Chicken manure digestate	Co-culture of green microalgal and actinomycetes consortium (gram-positive mycelial bacteria) Lab-scale flask culture	*Nocardia bhagyanarayanae* I-27 prompted a higher biomass (1.2 g/L), chlorophyll a (15.6 µg/mL) and lipid (20.8%) content in a co-culture with *Tetradesmus obliquus* using 25% diluted digestate	[75]
Not specified	Acidogenic effluents (fermented distillery wastewater and fermented dairy wastewater)	Mixotrophic culture, heterotrophic culture	Microalgae cultivation with mixotrophic mode using fermented distillery wastewater showed high biomass productivity in growth phase Microalgae cultivation with mixo- and heterotrophic mode using fermented dairy wastewater showed high lipid (34%) and neutral lipid (13%) content, respectively, in stress phase	[71]

### Bioprocesses for wastewater treatment

Wastewater or sludge such as sewage, domestic wastewater from households and industrial wastewater are usually generated through agricultural, industrial, domestic and commercial activities. The wastewater contains biological, chemical and physical pollutants, therefore, a proper wastewater treatment process is crucial to minimise the water pollution besides attaining environmental security. Looking towards this perspective, bioprocessing represents a potential wastewater treatment approach. In addition, wastewater reclamation with bioprocessing to produce value-added products is a crucial research field as wastewater contains vast amount of nutrients that is essential to nurture microbial culture (e.g., soluble and insoluble organic compounds which represent rich source of nitrogen, phosphorus and ammonium). The strategy of cultivating microorganisms using wastewater will promote the bioremediation of the wastewater in which reduces the cultivation cost and allows the production of many useful bio-based products (e.g., biopolymers, biofuels and health food supplements) and metabolites (e.g., proteins, lipids, carbohydrates and bio-actives) to be co-synthesised [14, 80–82]. Sarris et al. [83] evaluated the cultivation of *Saccharomyces cerevisiae* MAK-1 with olive mill wastewater treatment. A notable decolourisation and phenol removal efficiency ~ 63% and 34%, respectively, for the wastewater bioremediation were reported. Besides, the microbial culture enriched with the wastewater showed promising outcomes on the bioethanol and lipids production. Various researches have been conducted in this area, and there are still many ongoing investigations due to the potential of wastewater reclamation using bioprocesses. Various examples of bioprocesses incorporated with wastewater treatment and reclamation are presented in Table 3. Valorisation of waste into bioprocesses on the production of biopolymers and bio-lipids as well as bioprocesses for wastewater bioremediation can be represented as a sustainable and economical approach towards achieving a circular bioeconomy.

**Table 3:** Several bioprocesses integrated with wastewater bioremediation

Microbial strain	Type of wastewater	Results	Reference(s)
*Aspergillus oryzae*	Potato processing wastewater	COD removal efficiency = 91% Total soluble nitrogen removal efficiency = 98% Total soluble phosphorus removal efficiency = 97% Lipid concentration = 3.5 g/L	[84]
*Bjerkandera adusta* MUT 2295	Coloured wastewaters: textile industry wastewater, tannery industry wastewater and industrial dyes	*Bjerkandera adusta* MUT 2295 effectively degraded and detoxified most of the coloured wastewaters	[85]
Microalgae: *Chlorella sorokiniana* DBWC2 and *Chlorella* sp. DBWC7 Bacteria: *Klebsiella pneumoniae* ORWB1 and *Acinetobacter calcoaceticus* ORWB3 (co-culture of microalgae-bacteria consortium)	Raw dairy wastewater	COD removal efficiency = 90.49% Nitrate removal efficiency = 84.69% Biomass concentration = 2.87 g/L	[86]
*Chlorella vulgaris* FACHB-31	Mixed piggery-brewery wastewater	Ammonia removal efficiency = 100% TN removal efficiency = 96% TP removal efficiency = 90% COD removal efficiency = 93% Biomass concentration = 2.85 g/L	[87]
*Chlorella vulgaris* AG 30007 and *Pseudomonas putida* ATCC 17514 (co-culture of microalgae-bacteria consortium)	Municipal wastewater	COD removal efficiency = 86% Nitrogen removal efficiency = 78–85% Phosphorus removal efficiency = 54–65%	[88]
*Chlorella vulgaris* NIES-227 (co-culture of microalgae-bacteria consortium)	Sewage (activated sludge)	COD removal efficiency = 82.7% Nitrogen removal efficiency = 75.5% TP removal efficiency = 100% Biomass productivity = 343.3 mg/L/d The biomass produced showed a higher calorific value and protein content	[89]
*Micractinium* sp. IC-76	Municipal wastewater	Nitrogen removal efficiency = 96.4% Phosphorus removal efficiency = 77.8% Biomass productivity = 37.18 mg/L/d Lipid content = 36.29%	[90]
*Saccharomyces cerevisiae* MAK-1	Olive mill wastewater	Remarkable decolourisation (~ 63%) and phenol removal efficiency (~ 34% (w/w)) Co-production of bioethanol and lipids	[83]
*Scenedesmus* sp. (co-culture of microalgae-bacteria consortium)	Starch wastewater (anaerobic sludge)	Co-cultivation enhanced biohydrogen production and performed wastewater bioremediation COD removal efficiency = 80.5% Total nitrogen (TN) removal efficiency = 88.7% Total phosphorus (TP) removal efficiency = 80.1% Biohydrogen yield = 1508.3 mL/L Total lipid concentration = 0.36 g/L Energy conversion efficiency = 34.2%	[91]
Bacterial consortium ‘Bx’	Textile wastewater contains reactive dye	Maximum decolourisation rates = 88–97% Chemical oxygen demand (COD) removal efficiency = 95–98%	[92]
PHA-storing and filamentous bacteria	Municipal wastewater	COD removal efficiency = 70% COD_sol_ concentration removal efficiency = 60% (sol: soluble) Nitrogen removal efficiency = 24% Phosphorus removal efficiency = 46% Co-produced PHA	[93]
Microbial community	Lactate wastewater (obtained from cattle slaughterhouse)	COD removal efficiency = 12–30% Lactate removal efficiency = 54–99.8% Biohydrogen yield = 0.08–0.95 mol H_2_/mol lactate uptake (by dark fermentation process) Identified microbial = *Clostridium*, *Sporanaerobacter* and *Pseudomonas*	[94]
Sulphate reducing bacteria consortium	Real wastewater from Okhla industrial area effluent	Sulphate removal efficiency = 90% Chromium removal efficiency = 82.6% Cadmium removal efficiency = 86.6% Zinc removal efficiency = 54.09% Lead removal efficiency = 49.8% Nickel removal efficiency = 10.3% Oil and grease removal efficiency = 75%	[95]
Not specified (co-culture microbial consortium: microalgae, bacteria and other microscopic organisms)	Domestic sewage	TN removal efficiency = 72–83% TP removal efficiency = 100%	[96]
Not specified	Municipal biological wastewater	COD removal efficiency = 83% TN removal efficiency = 80% PHA content = 49%	[97]

## Strategies of waste biorefinery–circular bioeconomy towards solving the global issues

Global issues relating to the environment and food security are the defining problems of our time that have triggered the global awareness of the society. Much efforts have been made by different parties including the government, non-governmental organisations (NGOs), scientific communities and academia to resolve the problems progressively [4–6]. An unprecedented climate change (i.e., ever-changing weather patterns) can threaten the production of food crops which will cause a major setback in food sources. A speedy industrialisation and urbanisation are regarded as the major contributing factors on climate change as these processes release a high and unsafe level of GHGs mostly CO_2_ emissions to the atmosphere. The burning of fossil fuels is also among the most intimidating actions causing high GHG emissions [98–100]. A special report issued by Intergovernmental Panel on Climate Change (IPCC) in October 2018 stated that a global warming of 1.5 °C could trigger the negative impacts of climate change in terms of sea level rise and unsecure food production [6]. In this regard, waste biorefinery incorporated with circular bioeconomy represents a low carbon economy by involving CO_2_ sequestration which can resolve the global issues. Moreover, this strategy also signifies a sustainable and economical manner of waste disposal [7–9].

The valorisation of waste or side streams into bioprocesses for the production of value-added bioproducts such as biopolymers and biofuels could potentially replace the utilisation of fossil fuels as the production feedstock which ensures an ecologically friendly carbon flow. This approach is regarded as a waste-as-a-value, waste-to-wealth or zero-waste plan which would highly contribute as a decent, green, and low-cost waste disposal means. In addition, the bio-based products produced possess environmental benign properties such as non-toxicity, biodegradable and biocompatible that supports an eco-friendly campaign, and hence promoting a greener environment globally. Numerous environmental problems like global warming, water and environment pollution, waste disposal as well as natural resource depletion can then be unravelled. As an example, the development of bioplastics or biopolymers which substantially replace conventional petrochemical plastics can help to minimise plastic pollution that demonstrates adverse impacts in soil and marine ecosystem [101, 102]. Other than that, a great deal of research attention has been placed on improving the efficiency, effectiveness, and economic feasibility of wastewater treatment, and, therefore, water pollution can be conceivably addressed. The bioprocesses integrated with wastewater treatment (i.e., a type of biological wastewater treatment) have been proved to effectively bioremediate wastewater (e.g., sewage and industrial wastewater e). Besides that, wastewater reclamation for value-added bio-based products can be achieved by cultivating live microorganisms such as bacteria, algae and yeasts with wastewater [103]. To attain an energy security, bioenergy and biofuels has been produced from the microorganisms, and its production is non-dependent on petroleum feedstock. GHGs’ mitigation and carbon management can be achieved using biofuels for various purposes (e.g., transportation fuels). An economical manner of biofuels production (or microbial lipids synthesis) can then be accomplished by bioprocessing with a waste biorefinery [69, 104, 105].

Collectively, a waste biorefinery–circular bioeconomy strategy could ensure an energy–environmental security. Having an environmental security prompts a food security for the globe. Food security is of extremely importance to ensure an adequate supply of food resources for the increasing world population, and thus to avoid world hunger issue. Also, the quality of life and human health can be maintained through an environmental-food security. Hence the efforts of a circular bioeconomy will help to regenerate the good efficiency and prosperity in a life-long cycle without worry of the economic impacts of environmental, food and energy.

## Conclusions

A sustainable and eco-benign manner of waste disposal is critical to protect the environment and human health. In this regard, waste biorefinery exemplifies its potential. Valorisation of waste or side streams into bioprocessing to produce value-added bioproducts like biopolymers and bio-lipids remarkably advocate a sustainable circular bioeconomy. A circular bioeconomy which represents a low carbon economy by reducing GHGs footprint helps to resolve the global issues significantly such as environmental problems and food security. A waste biorefinery–circular bioeconomy strategy, therefore, holds great prospective for a sustainable green world and should be prompted.

## Data Availability

Not applicable.

## Abbreviations

BC: Bacterial cellulose
COD: Chemical oxygen demand
DHA: Docosahexaenoic acid
GHG: Greenhouse gas
HA: Hydroxyalkanoate
IPCC: Intergovernmental panel on climate change
LCL: Long chain length
MCL: Medium chain length
NGO: Non-governmental organisation
PHA: Polyhydroxyalkanoate
PHB: Poly(3-hydroxybutyrate)
PHBV: Poly(3-hydroxybutyrate-*co*-3-hydroxyvalerate)
PHV: Poly(3-hydroxyvalerate)
PUFA: Polyunsaturated fatty acid
SCL: Short chain length
SDG: Sustainable development goal
sol: Soluble
TN: Total nitrogen
TP: Total phosphorus
VFA: Volatile fatty acid
